# The Respiratory System of the *Arctocephalus australis* in Comparison to the Dog as a Land-Carnivore: Are There Adaptations to Marine Life?

**DOI:** 10.3390/ani13020199

**Published:** 2023-01-05

**Authors:** Ignacio Molpeceres-Diego, Rosario Martín-Orti, Juan-Pablo Loureiro, Carlos Tostado-Marcos, Enrique Tendillo-Domínguez, Inmaculada Santos-Álvarez, Pilar Pérez-Lloret, Juncal González-Soriano

**Affiliations:** 1 Fundación Mundo Marino, Av. X 157, San Clemente del Tuyú B7105, Provincia de Buenos Aires, Argentina; 2 Departamento de Anatomía y Embriología, Sección Departamental de Anatomía y Embriología Veterinaria, Facultad de Veterinaria, Universidad Complutense, Avenida Puerta de Hierro s/n, 28040 Madrid, Spain

**Keywords:** *Arctocephalus australis*, anatomy, respiratory system, land-carnivore, dog

## Abstract

**Simple Summary:**

It is normally recognized that anatomy is crucial for an improved knowledge of many physiological adaptations. In the case of marine mammals, their ability to dive for long periods of time is especially striking. They spend most of their lives in water, although in general terms, they behave and have characteristics very similar to those of land mammals. Our results demonstrate that, due to its capacity to stay under water, the respiratory apparatus of the South American fur seal shows specific characteristics. Yet, in general terms, being a carnivore seems to be the main characteristic of this species, and the adaptations to the aquatic environment being less important.

**Abstract:**

Marine mammals are divided into three groups, with similar adaptations resulting from their aquatic lifestyle: sirenians, pinnipeds, and cetaceans. The present work focused on the South American fur seal, or *Arctocephalus australis*, a carnivore included in the pinnipeds group. We assessed whether the anatomical features of the *Arctocephalus australis’* respiratory system are comparable to those of other land-carnivores or whether these individuals show anatomical adaptations related to their ability to dive or their breath-holding capacities. We studied 11 cadavers of *Arctocephalus australis*, which included adult (*n* = 2) and juvenile (*n* = 9) individuals, by anatomically dissecting their isolated entire respiratory system. Although it is generally similar to that in land-carnivores, we demonstrated that the *Arctocephalus australis*’s respiratory apparatus shows several specific characteristics. Therefore, our results are of great importance for clinical diagnostic and wildlife conservation purposes.

## 1. Introduction

Marine mammals have evolved from common ancestors that changed from a terrestrial life to an aquatic environment. Yet, aquatic life presents numerous challenges for those mammals originally adapted for life on land. Most of them develop their life, at least in part, in an aquatic environment; therefore, it is supposed that some of their morphological and physiological characteristics are linked to an organized evolution to marine-life adaptation.

However, despite all these changes, marine mammals still share some basic characteristics with terrestrial mammals, such as breathing through lungs, being warm-blooded, having fur (periodically in life), and producing milk to nurse their young [1,2,3]. As marine mammals do not breathe in water, modifications to the respiratory tract are needed to protect a system designed to function on land. Many of these adaptations have already been described, for example, valvular nostrils that exclude water, or an intranarial larynx in odontoceti that, while swallowing, further protects the respiratory tract from water inundation [4]. In addition, diving presents additional challenges, as ambient pressure rises with depth, potentially causing the lungs to collapse [5].

The present study focused on the South American fur seal or *Arctocephalus australis,* an otariid included in the taxonomic family of pinnipeds (Order Carnivora), such as the dog or the cat [6,7]. Surprisingly, although it is generally accepted that anatomy is crucial to a better understanding of different physiological adaptations [2], and although studies on the physiology of diving are numerous [1,8,9,10,11,12,13,14,15,16,17,18,19], only a small number of them analyze the anatomical characteristics of marine mammals [5,20,21,22,23,24], and even fewer are focused on the anatomy of the respiratory system [2,3,25,26,27].

In the South American fur seal, its capability to remain submerged for some time is well known. However, previous studies on the anatomy of the South American fur seal are scarce. Thus, the possible anatomical adaptations of its respiratory system are yet to be described. In other words, the question remains whether the respiratory system of the *Arctocephalus australis* is similar to that in other carnivores or if there are significant anatomical adaptations that allow the South American fur seal to dive.

In this work, we tried to answer this question. Thus, the objective of the present paper was to perform an anatomical, systematic, and descriptive study of the South American fur seal’s respiratory system and then compare it to the respiratory apparatus of another carnivore model, the dog.

## 2. Materials and Methods

The study was performed on 11 cadavers of the species *Arctocephalus australis*, including adult (*n* = 2) and juvenile (*n* = 9) individuals. All animals were found stranded on the coast and taken to the Rescue and Rehabilitation Center Fundación Mundo Marino (FMM), where they died. It is important to highlight that none of the individuals were euthanized. They were all frozen until their dissection. After thawing for a period of 24/48 h, animals were placed in dorsal recumbency and dissected by veterinarians.

As a first step, the skin and part of the blubber and muscle were removed. With a scalpel blade, we made a first incision under the chin and continued down the midline (*linea mediana ventralis*) to the xiphoid cartilage (*cartilago xiphoidea)*. To make the internal tissues visible, we extended the cut laterally along the costal arch.

Before cutting the ribs, we perforated the diaphragm. To open the thoracic cavity, we cut through each thoracic rib mid-articulation (*genu costae*), which is the cartilaginous flex point, and removed the rib cage..

For exposing the trachea and esophagus, the superficial musculature of the neck area (*regio collis ventralis*) was removed. Then, we made a triangular incision along the inner sides of the mandible body. The hyoid apparatus was cut, and the tongue was extracted through the intermandibular space. The respiratory system was then progressively separated.

As the present study included both juvenile and adult individuals, each with a different degree of development, the corresponding arithmetic means of the data obtained were calculated separately.

Pictures were taken with a Canon EOS 500D (Tokyo, Japan) camera equipped with an 18–55 mm lens.

As a complementary study, we prepared additional material:

- Sample 1. An exhaustive washout period of the trachea, bronchi and lungs was performed by leaking liquid for several days. Once cleaned, the empty ducts were filled by injecting compressed air.
- Sample 2. To obtain a template of the whole bronchial tree, expansive polyurethane was inserted through the trachea. Once polyurethane was expanded and cured, the sample was incubated with flies and larvae for tissue digestion to obtain the polymeric template of the lower respiratory tract.

## 3. Results

### 3.1. Nose and Nares

**Figure 1:**
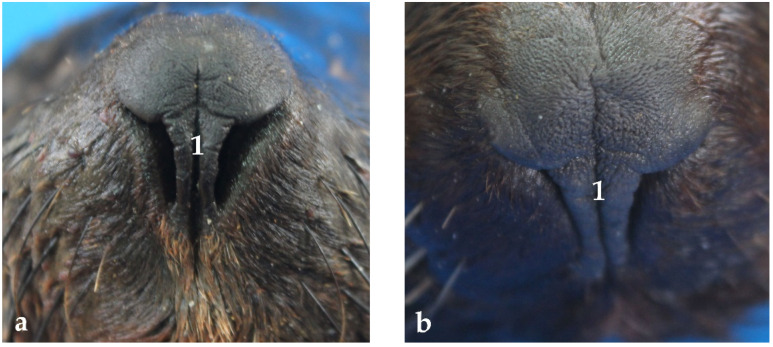
Frontal view of the nares: opened (**a**) and slightly closed (**b**). Note the presence of different furrows, (1) middle furrow or philtrum.

The nose is completely coated with hairy skin except the apex, where nares are placed and joined with the upper lip to give a higher functional mobility. The bones of the dorsal and lateral nasal walls are formed by the facial bones, except in the rostral region. This rostral region contains the dorsal and ventral lateral nasal cartilages. They are the bilateral extension of the dorsal and ventral borders of the cartilaginous nasal septum. The nares are placed in the nose apex. They are “boomerang”-shaped openings (Figure 1). The nose apex, the nasal mimetic muscles, and the nares are fixed by a cartilaginous skeleton. The surrounding skin, as well as the skin between the nares, is hairless and pigmented. It forms the nasal plane, which presents small superficial furrows. The middle furrow or philtrum extends all along the nasal plane, dividing it partially and not reaching the upper lip (Figure 1).

### 3.2. Nasal Cavity

The nasal cavity of the South American fur seal is narrow and long. It is divided into two halves (right and left) by the nasal septum. In both halves, the ventral part is bony, whereas the dorsal and rostral parts are cartilaginous. The perpendicular plate of the ethmoid bone and the vomer forms the bony portion. Caudally, it does not reach the lower part of the nasal cavity; thus, both nasal cavity halves are communicated. The cartilaginous segment expands from the perpendicular plate of the ethmoid bone to the incisive bones.

Caudally to the nasal cavity, the choanae connects the nasal cavity to the nasopharynx. Each nasal cavity is mainly filled by the nasal conchae (dorsal, middle, and ventral conchae), extending from the walls until nearly the nasal septum. The dorsal nasal conchae are relatively long and narrow, with a smooth surface, whereas the ventral is shorter and wider and forms little parallel folds (*plicae parallelis*). The medial nasal conchae are placed in between the other two.

The conchae divide each nasal cavity into subsequent parts called meatus. The dorsal nasal meatus is narrow and short. It is placed between the dorsal nasal conchae and the roof of the nasal cavity. The middle nasal meatus is located between the dorsal and ventral nasal conchae. It is also short and narrow. The ventral nasal meatus is longer than the other two. It extends from the bottom of the nasal cavity to the nasal ventral conchae and leads into the nasopharynx. Therefore, the ventral meatus represents a direct passage of air into the larynx and lower respiratory tract. The common nasal meatus is the narrow space between the nasal septum and the conchae, and it continues laterally with the other meatuses.

**Figure 2:**
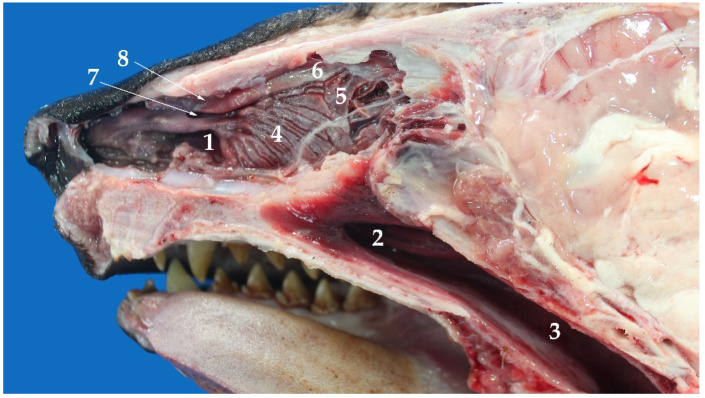
Lateral view of the left nasal cavity: (1) ventral nasal meatus; (2) choana; (3) nasopharynx; (4) ventral nasal conchae with parallel folds (*plicae parallelis*); (5) middle nasal conchae; (6) dorsal nasal conchae; (7) middle nasal meatus; (8) dorsal nasal meatus.

In fact, the choanae represent a direct path from the nasal cavity into the nasopharynx. They are bounded rostrally by the horizontal portion of the palatine bone, laterally by the pterygoid bone and the perpendicular plate of the palatine bone, and caudally by the vomer bone. Interestingly, there are no paranasal sinuses in the *Arctocephalus australis* (Figure 2).

### 3.3. Pharynx

The pharynx is divided into the oropharynx, nasopharynx, and laryngopharynx. The most noteworthy detail is the length of the soft palate, with a significant amount of peripharyngeal soft tissue.

### 3.4. Larynx

**Figure 3:**
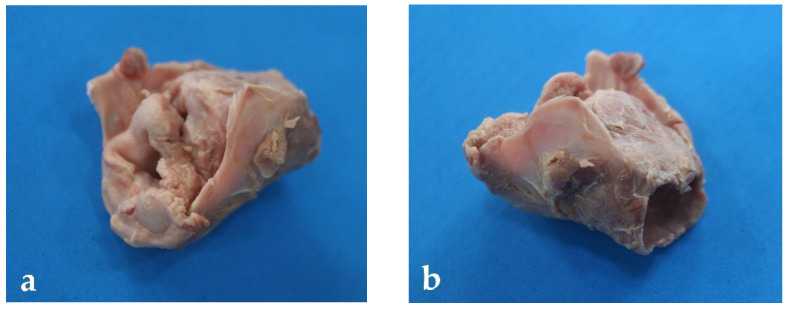
Larynx of *Arctocephalus australis*: (**a**) oblique view of the rostral larynx; (**b**) oblique view of the caudal larynx.

**Figure 4:**
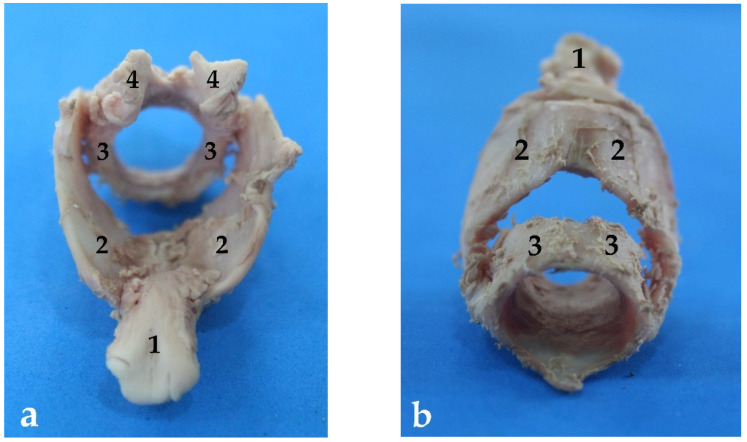
Larynx cartilaginous structure of *Arctocephalus australis*: (**a**) rostral view, (**b**) caudal-ventral view, (1) epiglottis, (2) thyroid cartilage, (3) cricoid cartilages, (4) arytenoid cartilages.

The larynx is relatively short and wide (Figure 3). It is located underneath the skin, ventral to the CI and CII vertebral body. It is dorsally related to the pharynx and the esophagus, ventrally to the sternohyoid muscle, and laterally to the sternothyroid muscle and the mandibular gland. The cartilages are cricoid, thyroid, arytenoid, and epiglottis (Figure 4).

### 3.5. Trachea

**Figure 5:**
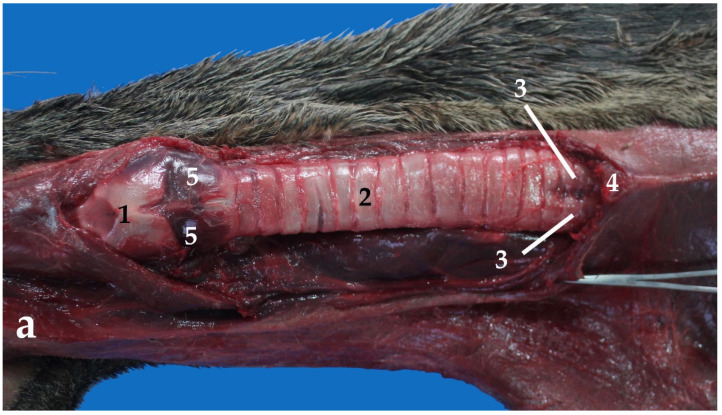

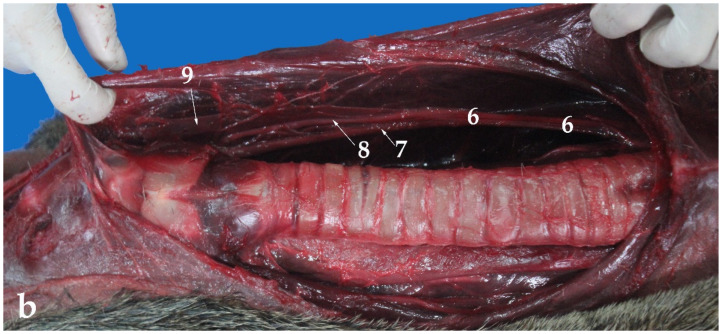
Dorsal view of the neck: (**a**) superficial view, (**b**) deeper view with the carotid sheath in detail, (1) larynx; (2) trachea; (3) bronchi; (4) sternal manubrium; (5) thyroid gland; (6) carotid sheath (opened); (7) common carotid artery; (8) vagosympathetic trunk; (9) internal jugular vein.

The trachea is a membranous and cartilaginous flexible duct that forms the proximal part of the lower respiratory airways. It extends from the larynx to the tracheal bifurcation (*bifurcate tracheae*). In young individuals, the trachea reaches the area below the sternal manubrium. On the other hand, it does not go that far in adults. Additionally, in adults, the trachea splits into two main bronchi (left and right) immediately cranial to the tip of the manubrium (Figure 5a). In the South American fur seal, the trachea shows only a cervical portion, whereas it is not possible to identify a thoracic portion. The tracheal length average is around 8 cm in juveniles and 13 cm in adults, whereas the width in juveniles has a value of approximately 2 cm and 3 cm in adults (Table 1).

**Figure 6:**
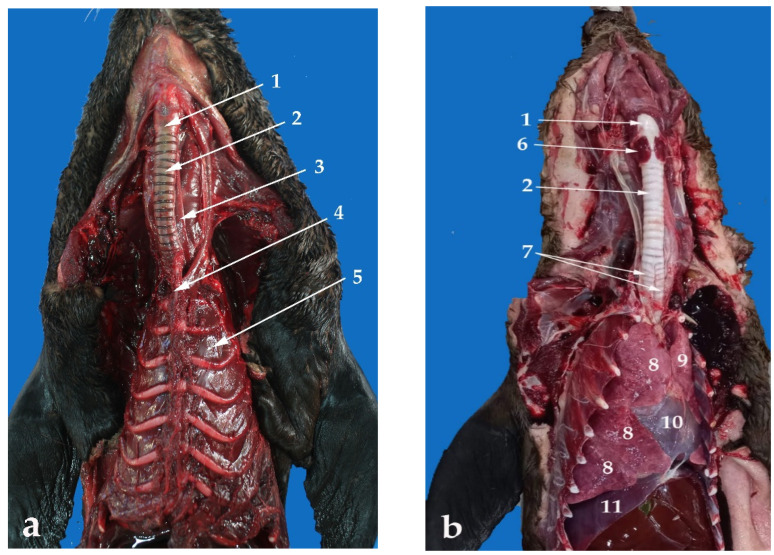
Ventral view of the neck and the thorax (**a**) and the neck and thoracic and abdominal cavities (**b**): (1) larynx; (2) trachea; (3) esophagus; (4) sternal manubrium; (5) thorax; (6) thyroid gland; (7) main bronchi; (8) right lung; (9) left lung; (10) heart; (11) diaphragm.

As mentioned before, the trachea of the *Arctocephalus australis* has only a cervical portion, practically in the midline (Figure 5 and Figure 6). The common carotid artery, the internal jugular vein, and the vagosympathetic trunk are included in the carotid sheath. The recurrent laryngeal nerve is located on the dorsolateral portions of the trachea, either close to its dorsolateral side, in the cranial half of the neck, or close to its lateral side, in the caudal half of the neck. (Figure 5b). The lobes of the thyroid gland and the small parathyroid glands are related to the lateral aspects of the cranial part of the trachea. The trachea is also related to different lymphatic structures and to the external jugular vein, at the thoracic entrance.

**Figure 7:**
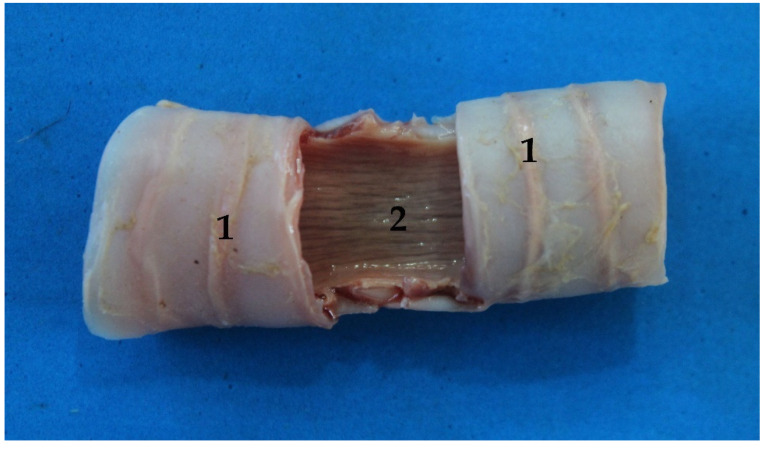
Trachea section, ventral view: (1) cartilaginous rings; (2) membranous part.

There are 12–14 incomplete cartilaginous rings, U-shaped, that constitute the trachea of the South American fur seal, plus one more as a transition between the trachea and the bronchi (Table 1). Their dorsal part is free but attached by tracheal muscles that form the dorsal part of the tracheal wall. It is known as the membranous portion (Figure 7).

The diameter of the tracheal lumen is 1.5 cm in juveniles and 2.5 cm in adults, whereas the tracheal circumference is about 6 cm in the former and 6.8 cm in the latter (Table 1).

### 3.6. Bronchi

**Figure 8:**
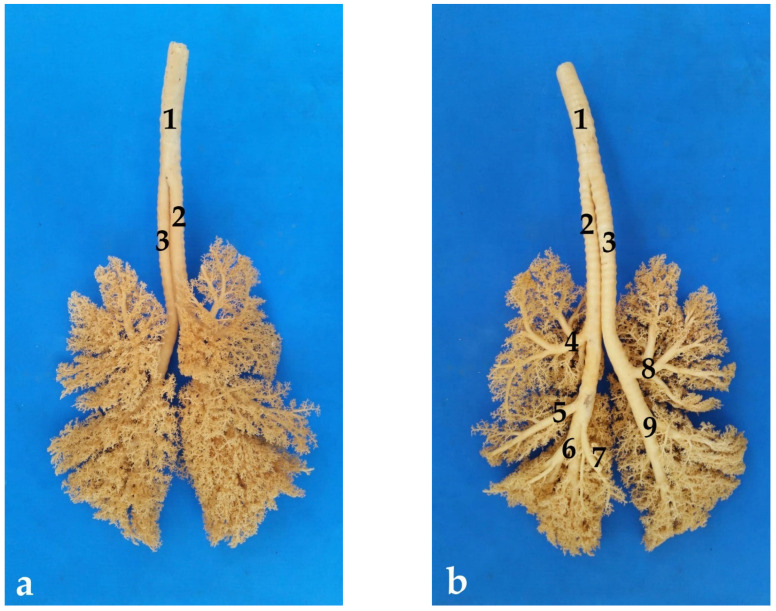
Polyurethane bronchial tree: (**a**) dorsal view; (**b**) ventral view; (1) trachea; (2) right main bronchus; (3) left main bronchus; (4) right cranial lobar bronchus; (5) right mid lobar bronchus; (6) right caudal lobar bronchus; (7) accessory lobar bronchus; (8) left cranial lobar bronchus; (9) left caudal lobar bronchus.

As previously described, the trachea of the South American fur seal early bifurcates into the two main bronchi at the level of the caudal third of the neck, practically at the sternal manubrium. The right and left main bronchi are in close contact with some of the great thoracic vessels, such as the cranial vena cava or the aortic arch. The first branch is the right main bronchus (Figure 8).

After traveling about 8 cm in juveniles and about 14 cm in adults, a second bronchial branch emerges. It is the right cranial lobar bronchus, which ventilates the cranial lobe of the right lung. This right main bronchus continues traveling about 5 cm more, inside the thoracic cavity, until the lung hilus, where the other three branches of the right main bronchus arise almost simultaneously.

The first of these three branches is the so-called right middle lobar bronchus, which ventilates the middle lobe of the right lung. This bronchus extends throughout the entire middle lobe, branching into several collateral bronchi (*bronchi segmentalis*). Immediately after the right middle lobar bronchus, the caudal right lobar bronchus emerges, which ventilates the caudal right lobe. The right accessory lobar bronchus appears practically at the same level, which enters the accessory lobe of the right lung.

The left main bronchus gives rise only to two branches, both located at the level of the pulmonary hilus. This second left bronchial generation arises to about 11 cm from the tracheal bifurcation in juveniles and approximately 16 cm in adults, cranially to the triple branching of the right main bronchus. The left main bronchus branches into two secondary bronchi: the left cranial bronchus and the left caudal bronchus. The left cranial bronchus ventilates the left cranial lobe, while the left caudal bronchus ventilates the left caudal lobe. Each of these secondary branches divide further into tertiary bronchi or segmental bronchi, which form the bronchioles (arbor alveolaris), ending in the alveolar sacs (Figure 8).

**Figure 9:**
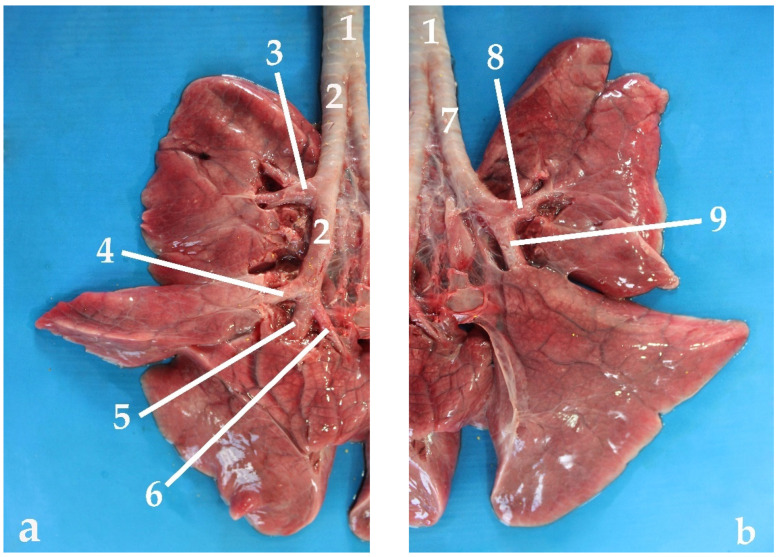
Ventral view of the lung: (**a**) right lung; (**b**) left lung; (1) trachea; (2) right main bronchus; (3) right cranial lobar bronchus; (4) right mid lobar bronchus; (5) right caudal lobar bronchus; (6) accessory lobar bronchus; (7) left main bronchus; (8) left cranial lobar bronchus; (9) left caudal lobar bronchus.

As mentioned before, the right main bronchus is slightly longer than the left one (Figure 9), which means that it has more cartilaginous rings, between 16 and 18 until the exit of the first branch of the right cranial bronchus, and another 7 or 8 more rings before the triple branching described previously (Table 2). The left main bronchus has between 19 and 22 cartilaginous rings up to the region where it divides into the two left secondary bronchi (Table 2). From this point, the diameter and width of the bronchi decrease accordingly until the alveolar sacs (Table 2). The cross-sectional area of each bronchus is approximately half of the tracheal area. Thus, the total cross-sectional area of the two primary bronchi is practically equal to the cross-sectional area of the trachea.

### 3.7. Lungs

**Figure 10:**
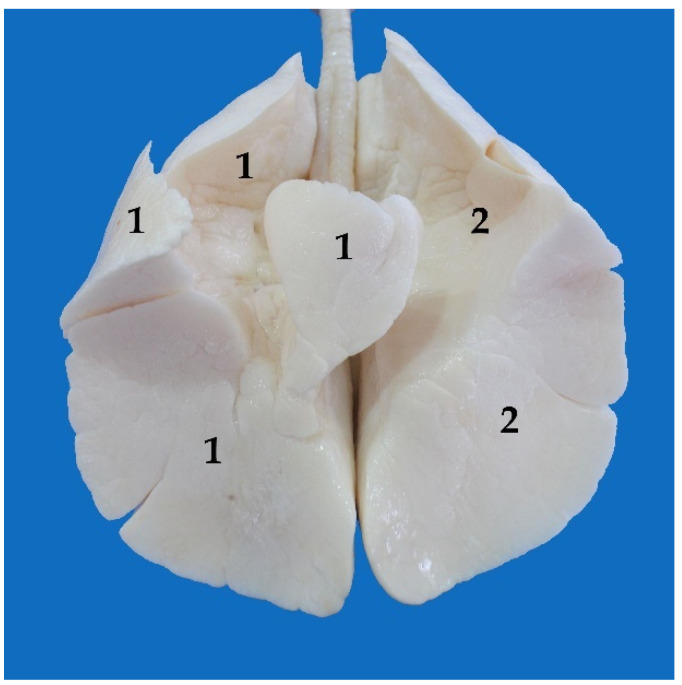
Ventral view of the lungs, washed with water and filled with compressed air: (1) right lung; (2) left lung.

**Figure 11:**
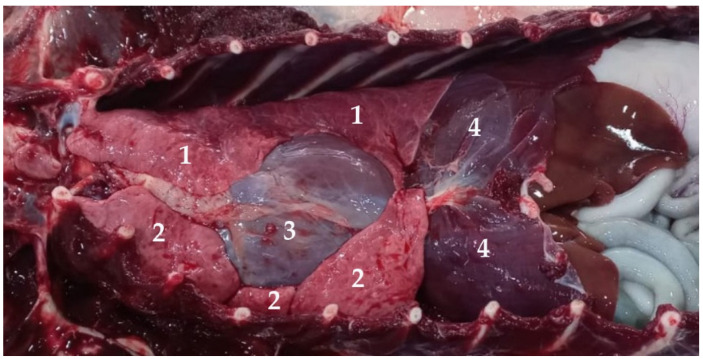
Ventral view of the thoracic cavity of *Arctocephalus australis*: (1) left lung; (2) right lung; (3) heart; (4) diaphragm.

There are two lungs, one right and one left (Figure 10), that occupy the largest part of the thoracic cavity (Figure 11). In the case of the South American fur seal, they approach the level of the 9th rib. Each lung is covered by the pulmonary pleura and invaginated in the ipsilateral pleural sac, to which it is attached by the pulmonary ligament. Inside this pleural sac, the lung can move freely.

**Figure 12:**
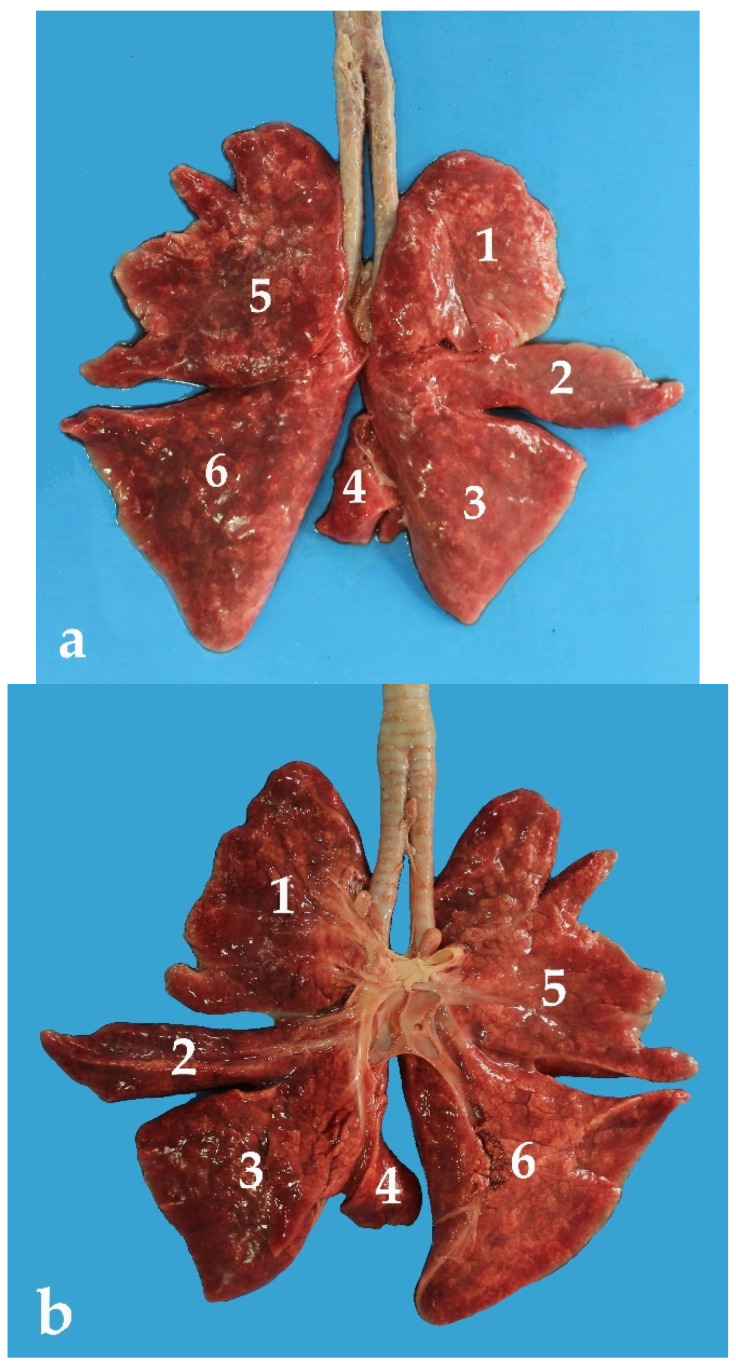
Lungs of *Arctocephalus australis*: (**a**) dorsal view; (**b**) ventral view; (1) right cranial lobe; (2) right middle lobe; (3) right caudal lobe; (4) right accessory lobe; (5) left cranial lobe; (6) left caudal lobe.

**Figure 13:**
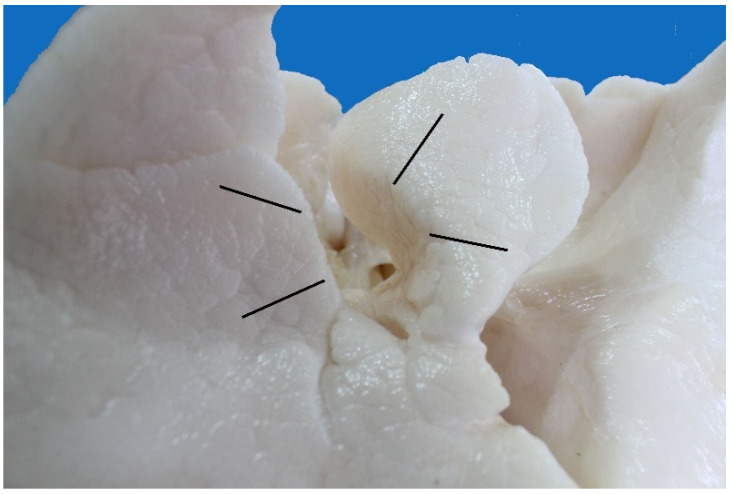
Detail of the groove formed by the accessory lobe for the passage of the caudal vena cava and the right phrenic nerve.

The lungs of the *Arctocephalus australis* are divided into lobes by deep interlobar fissures. In other words, these lungs show a well-defined lobulation, the patter of which is as follows (Figure 12):

- Right lung: It has four differentiated lobes, the first being the cranial lobe. Next, and separated from the cranial lobe by a deep interlobar fissure, is the middle lobe, with an elongated and pyramidal shape. Caudally to the middle lobe is the caudal lobe, with an expanded fan-like shape. The *Arctocephalus australis* has a fourth lobe in the right lung, the accessory lobe. It is located between both lungs, specifically in the caudal mediastinum (Figure 12). Ventrally, this accessory lobe has a groove for the passage of the caudal vena cava and the right phrenic nerve (Figure 13).
- Left lung: The left lung shows one pair of lobes. The first is the cranial lobe, which has an irregular surface. It is divided into two parts by a short fissure. The caudal lobe is large and has scarce subdivisions. Interestingly, its shape is very similar to that of the caudal right lobe (Figure 12).

**Figure 14:**
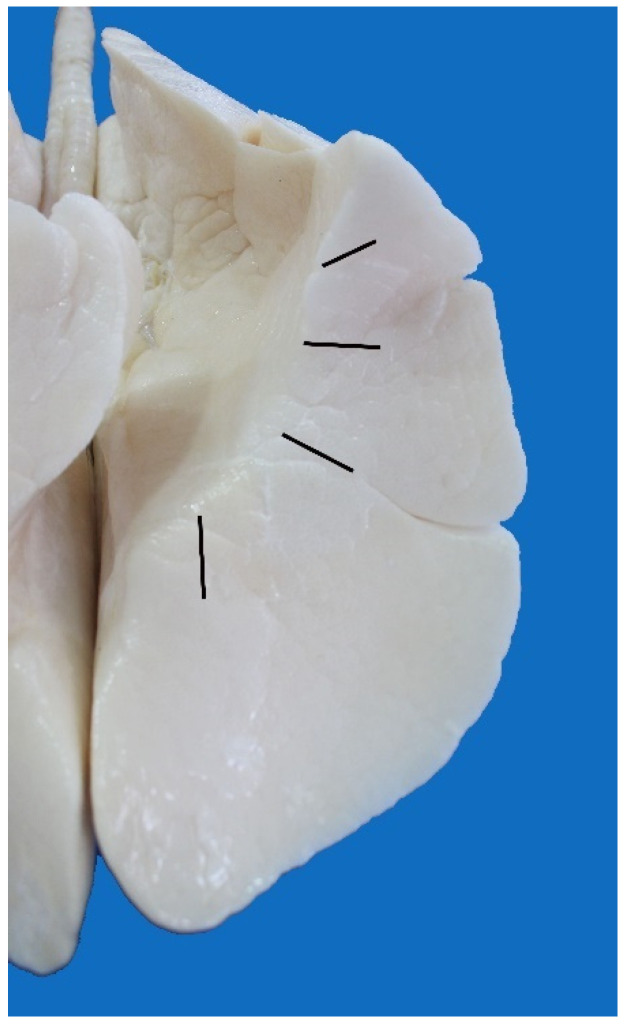
Detail of the cardiac impression in the left lung.

The different parts of both lungs can be described as follows:

1. Base, or diaphragmatic surface of the lung: It is concave as it overlaps the convex thoracic surface of the diaphragm. It is limited by the basal border.
2. Apex: It is free, sharpened, and laterally flattened. It fills the space of the pleural dome.
3. Two surfaces: (A) The costal surface: Lateral, larger, smooth, and convex. It is in contact with the inner surface of the lateral thoracic wall. (B) The medial surface: It is smaller than the costal and is related to the mediastinum and the mediastinal structures. It presents a deep depression formed by the heart and its pericardium, the cardiac impression. The cardiac impression is deeper in the left lung (Figure 14).
4. Three distinct borders (ventral, dorsal, and basal): (A) Ventral border: It is sharp and irregular. It occupies the costomediastinic sinus and presents the cardiac notch. The ventral border of the left lung is slightly greater than the one of the right lung (Table 3). (B) Basal border: The basal border separates the base (or diaphragmatic surface) from the costal and medial surfaces. As in the case of the ventral border, the basal border of the left lung has a greater extension than in the right lung (Table 3). (C) Dorsal border: It is thick and rounded. It forms the dorsal boundary between the costal surface and the medial surface. Contrary to what happens in the ventral and basal borders, the dorsal border of the right lung is the one with a greater length (Table 3).

The maximum width of both lungs is also different, and the values of the right side are slightly lower (Table 3). 

## 4. Discussion

Marine mammals, which live most of their lives in the ocean or in an aquatic environment, have typical characteristics of all mammals. Thus, in the case of their respiratory system, it also consists of the same parts, starting with the nose and nares and ending with the lungs.

It is important to remark that, in general terms, knowledge of anatomy helps to understand any physiological phenomenon. Surprisingly, and although they could provide key insights into physiological responses to dive, in the case of the *Arctocephalus australis*, previous reports on the anatomy of its respiratory system are scarce [2]. For example, the depths to which these animals can dive and the length of time they remain submerged have intrigued researchers for decades. Unlike what happens with land mammals, *Arctocephalus australis* has enough oxygen to perform long dives and is able to avoid pressure-related diseases from repetitive and deep diving. The question that arises is if there are anatomical differences between its respiratory apparatus and that of dogs, as a typical model of land-mammal, or if on the contrary, this species is able to live in the water environment without special anatomical adaptations.

As mentioned before, the respiratory apparatus of the South American fur seal starts with the nose and the nares. The nares have a greater mobility compared to other mammal species with similar nose physiognomy. They can intentionally be totally closed or even expand to double their size as compared to resting conditions [5]. Thus, the South American fur seal has the ability to open or close the nares as if they were valves to avoid a massive water uptake through the airways while diving. Additionally, they can increase the air expelled per unit time when approaching the surface [5]. In addition, contrary to what happens in other mammals such as the dog, the philtrum divides the nasal plane partially, as it does not reach the upper lip [6]. The nasal septum is also significantly shorter than in dogs or cats [6], which facilitates different pathologies affecting the upper respiratory tracts [28]. On the contrary, the nasal cartilage is similar to that of the dog [6].

There are four groups of bone cavities connected to the nasal cavity in land mammals: the paranasal sinuses. These sinuses act synergistically with the nasal cavity to warm and humidify the inhaled air during breathing. Pinnipeds, other marine mammals, have lost these paranasal sinuses due to the high pressures they face under water. These structures are too fragile to resist these high pressures. Thus, in the case that these structures remained in pinnipeds, they would fracture easily [18]. As in phocids, the soft palate of the *Arctocephalus australis* is much longer than that of the dog, with a significant amount of peripharyngeal soft tissue. This anatomical characteristic is of great clinical interest, taking into consideration the difficulties these marine species have for intubation [29]. In contrast, the larynx of the South American fur seal is practically like that of other carnivores [6]. Thus, the laryngeal cartilages are similar in shape and size to those of the dog.

As shown by other authors, tracheal length is directly related to the need of determined speed air flows. When analyzing the functional implications of this fact, it is important to compare the tracheal width to its length, since the ability to exchange large air volumes per unit time is directly related to the absolute diameter/length ratio [25]. This seems to be the reason why cetaceans have short and wide tracheas with a large pulmonary ventilations volume (80–90%) in shorter time, which is the opposite of what happens in land-carnivores, as these species do not have this need [2]. The *Arctocephalus australis* trachea is longer than in cetaceans, with an early bifurcation, but it is shorter than that in dogs and cats. This anatomical difference is probably related to its diving adaptation and its distinct ventilation need in comparison to other marine species [30].

Mammals show variations in the bronchial branching pattern, which is of great importance when analyzing their functional implications. Thus, their bronchial tree consists of a sequence of branches, whose number, generation, and distribution vary according to the different mammalian species. In summary, the branching pattern is similar to that in the dog [31,32].

As previously shown in these species, the cross-sectional area of each bronchus is approximately half of the tracheal area. Thus, the total cross-sectional area of the two primary bronchi is practically equal to the cross-sectional area of the trachea [2]. According to our results, this is also the case for the *Arctocephalus australis*, which, due to the early bifurcation of its trachea, has extremely long bronchi in comparison to other species of land mammals [6,7].

Interestingly, the *Arctocephalus australis* bronchial tree shows differences compared to other marine mammals, such as certain dolphin species. These animals have developed a third bronchus or tracheal bronchus. This bronchus ventilates the most cranial part of the right lung, independently of the main right bronchus entry [26,27].

The explanation could be in the South American fur seal lifestyle, as this species alternates land and water environments. They do not need rapid ventilation, as is the case in cetaceans, and neither live constantly on land. This could also be the reason why the *Arctocephalus australis* has a short trachea and long bronchi, especially when compared to land mammals, whose ventilation demands are different [25].

Concerning the lungs, our results showed that the lobulation pattern is very similar to that in other species of land mammals, such as the dog or the cat [6,7]. Surprisingly, the lungs of the South American fur seal are different from those of cetaceans, which show unilobed lungs [25,26,33]. This could be related to the ability of these species to mobilize large volumes of air at high speeds in short intervals of time; meanwhile, pinnipeds do not have that need as they spend more time on land [33].

As it has been previously shown, the lung lobulation of land mammals, with a greater absorption surface, is able to better bear internal impacts during terrestrial locomotion [26]. We think that this could be the case for the South American fur seal. However, as in other species of marine mammals, the lung microanatomy of this species has thicker and firmer connective tissue partitions in comparison to land mammals, which is a clear adaptation to the aquatic environment. This greater development of the septa forms a more rigid and resistant lung support stroma, giving the parenchyma a greater resistance to lung collapse that could occur during diving [2].

In summary, the lungs of the South American fur seal show their own differential characteristics, as they are a mixture of those of other mammal species, either marine or terrestrial

## 5. Conclusions

The anatomical characteristics of the South American fur seal’s respiratory apparatus are very similar to those observed in other land-carnivores, such as the dog. 

Nevertheless, this species exhibits several differences with other land-carnivores, of course resulting from their diving ability. Examples of this are its capacity to open or close the nares and the early bifurcation of the trachea, which does not have a thoracic portion.

There are also interesting differences between the lungs of the South American fur seal and other marine mammals, which is probably related to their different lifestyle and ventilation demands.

The conclusion is that, besides these differences, being a carnivore seems to be the critical characteristic defining the respiratory apparatus of the South American fur seal, even though there are clear differential anatomical details to facilitate its adaptation to the aquatic environment.

## Figures and Tables

**Table 1:** Morphometric data of the trachea from the 11 individuals of *Arctocephalus australis* investigated in the present study. A: adult; J: juvenile.

ID	Age Group	Sex	Length (cm)	Width (cm)	Lumen Tracheal Diameter (cm)	Tracheal Circumference (cm)	N° Cartilaginous Rings
M9719	J	F	6.9	1.6	1.2	5.4	12 + 1
M7419	J	M	7.3	1.7	1.3	5.6	12 + 1
M7019	J	M	7.8	1.9	1.4	5.9	13 + 1
M8419	J	F	8	1.8	1.4	6	14 + 1
M8319	J	M	8	2	1.5	6	13 + 1
M10019	J	F	8.1	2	1.5	6.1	12 + 1
M7919	J	M	8.3	2	1.5	6.2	12 + 1
M8219	J	F	8.7	2.1	1.6	6.2	12 + 1
M10419	J	M	9.1	2.2	1.7	6.2	12 + 1
M7319	A	F	12.8	2.7	2.4	6.5	13 + 1
M9919	A	F	14	3	2.5	6.7	12 + 1
Mean	J	-	8	1.9	1.5	6	12–14
Mean	A	-	13.4	2.9	2.5	6.6	12–14

**Table 2:** The morphometric data of the bronchi from the 11 individuals of *Arctocephalus australis* investigated in the present study. A: adult; J: juvenile.

ID	Age Group	Sex	Length (cm)		Width (cm)	Lumen Bronchus Diameter (cm)	Bronchus Circumference (cm)	N° Cartilaginous Rings	
			Right	Left				Right	Left
M9719	J	F	6.4 + 4.7	9.2	1.1	0.9	3.5–3.2	17 + 8	20
M7419	J	M	6.8 + 5	9.5	1.1	1	3.7–3.5	18 + 8	22
M7019	J	M	7 + 5	9.8	1.1	1	3.7–3.5	18 + 7	21
M8419	J	F	7 + 5	10	1.1	1	3.7–3.5	16 + 7	19
M8319	J	M	7.4 + 5.1	10.2	1.1	1	3.7–3.5	18 + 7	21
M10019	J	F	8.4 + 5.2	10.6	1.1	1	3.8–3.6	17 + 8	20
M7919	J	M	9 + 5.2	11	1.2	1.1	3.8–3.6	18 + 8	21
M8219	J	F	9.3 + 5.4	11.8	1.2	1.1	3.9–3.7	16 + 7	20
M10419	J	M	9.8 + 5.4	12.4	1.3	1.2	3.9–3.7	17 + 7	20
M7319	A	F	13 + 6	15.9	1.4	1.3	4.1–3.9	17 + 8	21
M9919	A	F	14 + 6.4	17.6	1.6	1.5	4.3–4	18 + 8	22
Mean	J	-	7.9 + 5	10.5	1.1	1	3.7	16–18 + 7/8	20–22
Mean	A	-	14 + 6.2	16.7	1.5	1.4	4.2	16–18 + 7/8	20–22

Width, lumen bronchus diameter, and bronchus circumference are equal in both bronchi, right and left.

**Table 3:** Morphometric data of the lungs from the 11 individuals of *Arctocephalus australis* investigated in the present study. A: adult; J: juvenile.

ID	Age Group	Sex	Length (cm)						Maximum Width	
			Dorsal Border		Ventral Border		Basal Border		(cm)	
			Right	Left	Right	Left	Right	Left	Right	Left
M9719	J	F	20.1	19.3	12.8	13.9	10.7	13.6	12.6	11.3
M7419	J	M	20.5	20	13	14.3	11	14	12.9	11.8
M7019	J	M	21.6	21	13.4	15	11.3	15.3	13.2	12.1
M8419	J	F	22.6	21.3	13.7	15.5	11.6	15.5	14	12.8
M8319	J	M	23.3	22	14.2	16.3	11.9	15.7	14.3	13
M10019	J	F	24	22.9	14.7	16.5	12.1	16.3	14.5	13.4
M7919	J	M	25.8	24.2	15	16.7	12.6	16.7	15.1	13.8
M8219	J	F	27.7	26	16.8	18.2	13.7	17.7	16.4	15.2
M10419	J	M	28	26.2	17.2	19	14.1	18	18	16.7
M7319	A	F	33	30.5	23.5	25.7	19.6	24.5	24	22.6
M9919	A	F	35	32.7	24.5	26.3	20.1	25	24.7	23
Mean	J	-	23.7	22.5	14.5	16.1	12.1	15.8	14.5	13.3
Mean	A	-	34	31.6	24	26	19.8	24.7	24.3	22.8

## Data Availability

Not applicable.
